# Supplementary material for: Somatic hypermutation analysis for improved identification of B cell clonal families from next-generation sequencing data
Source: PLoS Comput Biol. 2020 Jun 23;16(6):e1007977. doi: 10.1371/journal.pcbi.1007977 (PMC7347241; doi:10.1371/journal.pcbi.1007977)

Somatic hypermutation analysis for improved identification of B cell clonal families from next-generation sequencing data

Nima Nouri[1,2] and Steven H. Kleinstein[1,2,3,*]

[1]Department of Pathology, Yale School of Medicine, New Haven, CT 06511, USA.
[2]Center for Medical Informatics, Yale School of Medicine, New Haven, CT 06511, USA.
[3]Interdepartmental Program in Computational Biology and Bioinformatics, Yale University, New Haven, CT 06511, USA.

*To whom correspondence should be addressed.
Contact: steven.kleinstein@yale.edu

S1 Fig: The global metrics of the BCR simulated repertoires, including: (1) junction length distribution, (2) distance-to-nearest distribution, (3) clonal relative abundance distribution, (4) clone size distribution, (5) mutation frequency distribution, (6) number of clones per VJℓ-group, (7) average pair-wise SHM for clone, and (8) negative-control test (comparing pair-wise SHM sharing rate among real clones and a set of artificial clones generated by randomly sampling sequences across known clones).

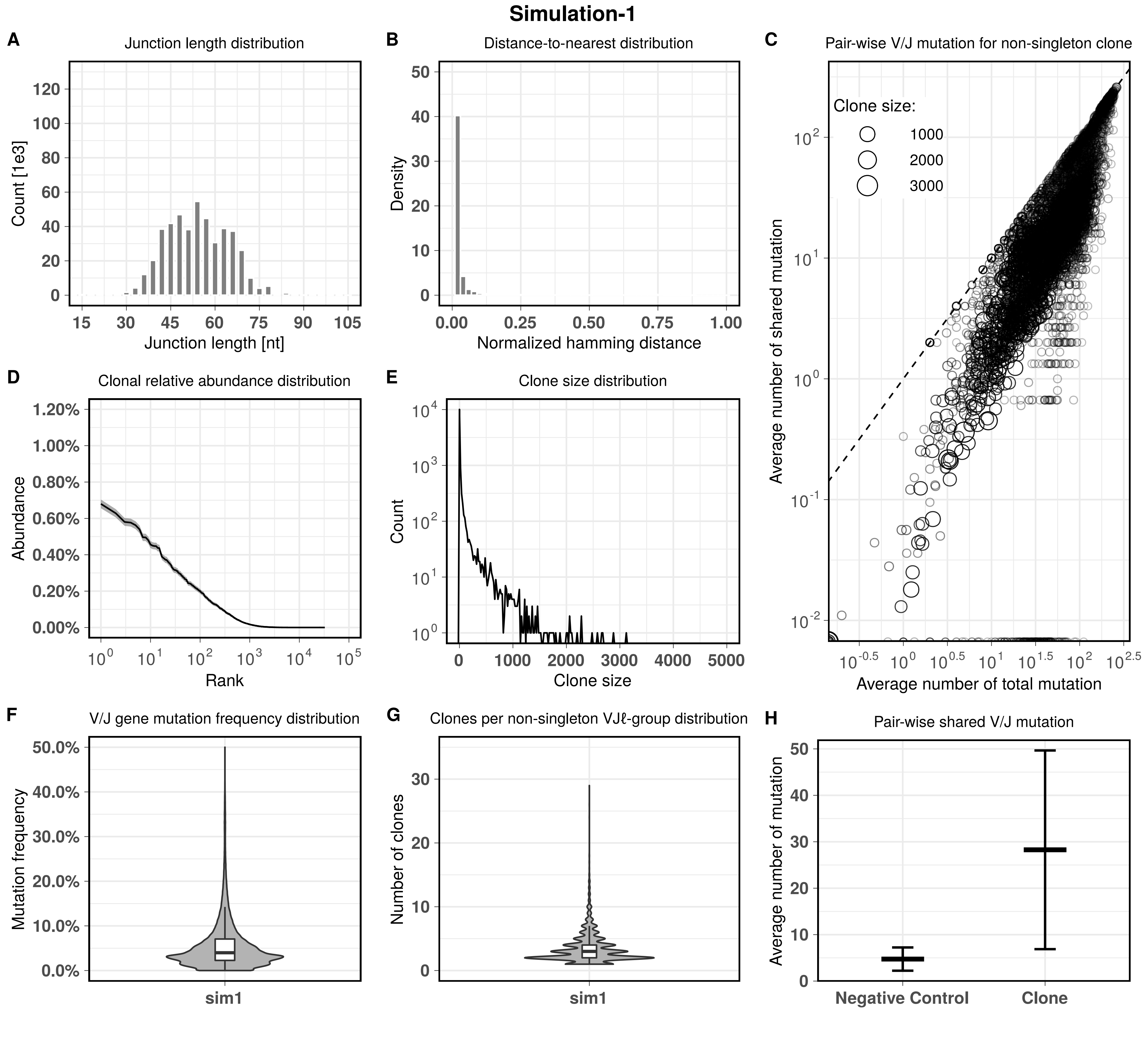
Simulation-1
A
Junction length distribution
Count [1e3]
Junction length [nt]
B
Distance-to-nearest distribution
Density
Normalized hamming distance
C
Pair-wise V/J mutation for non-singleton clone
Clone size:
1000
2000
3000
Average number of shared mutation
Average number of total mutation
D
Clonal relative abundance distribution
Abundance
Rank
E
Clone size distribution
Count
Clone size
F
V/J gene mutation frequency distribution
Mutation frequency
sim1
G
Clones per non-singleton VJℓ-group distribution
Number of clones
sim1
H
Pair-wise shared V/J mutation
Average number of mutation
Negative Control
Clone

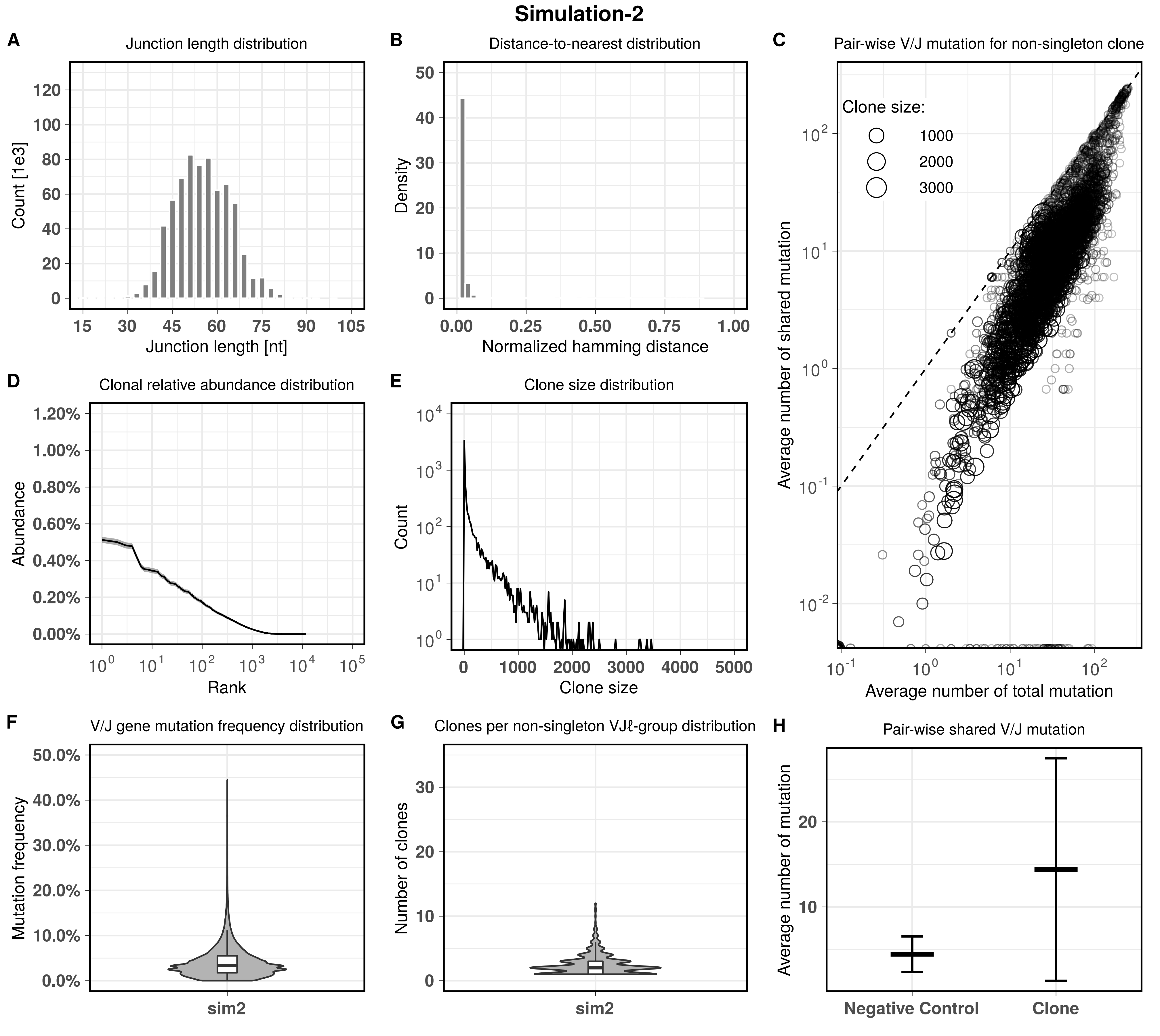
Simulation-2
A
Junction length distribution
Count [1e3]
Junction length [nt]
B
Distance-to-nearest distribution
Density
Normalized hamming distance
C
Pair-wise V/J mutation for non-singleton clone
Clone size:
1000
2000
3000
Average number of shared mutation
Average number of total mutation
D
Clonal relative abundance distribution
Abundance
Rank
E
Clone size distribution
Count
Clone size
F
V/J gene mutation frequency distribution
Mutation frequency
sim2
G
Clones per non-singleton VJℓ-group distribution
Number of clones
sim2
H
Pair-wise shared V/J mutation
Average number of mutation
Negative Control
Clone

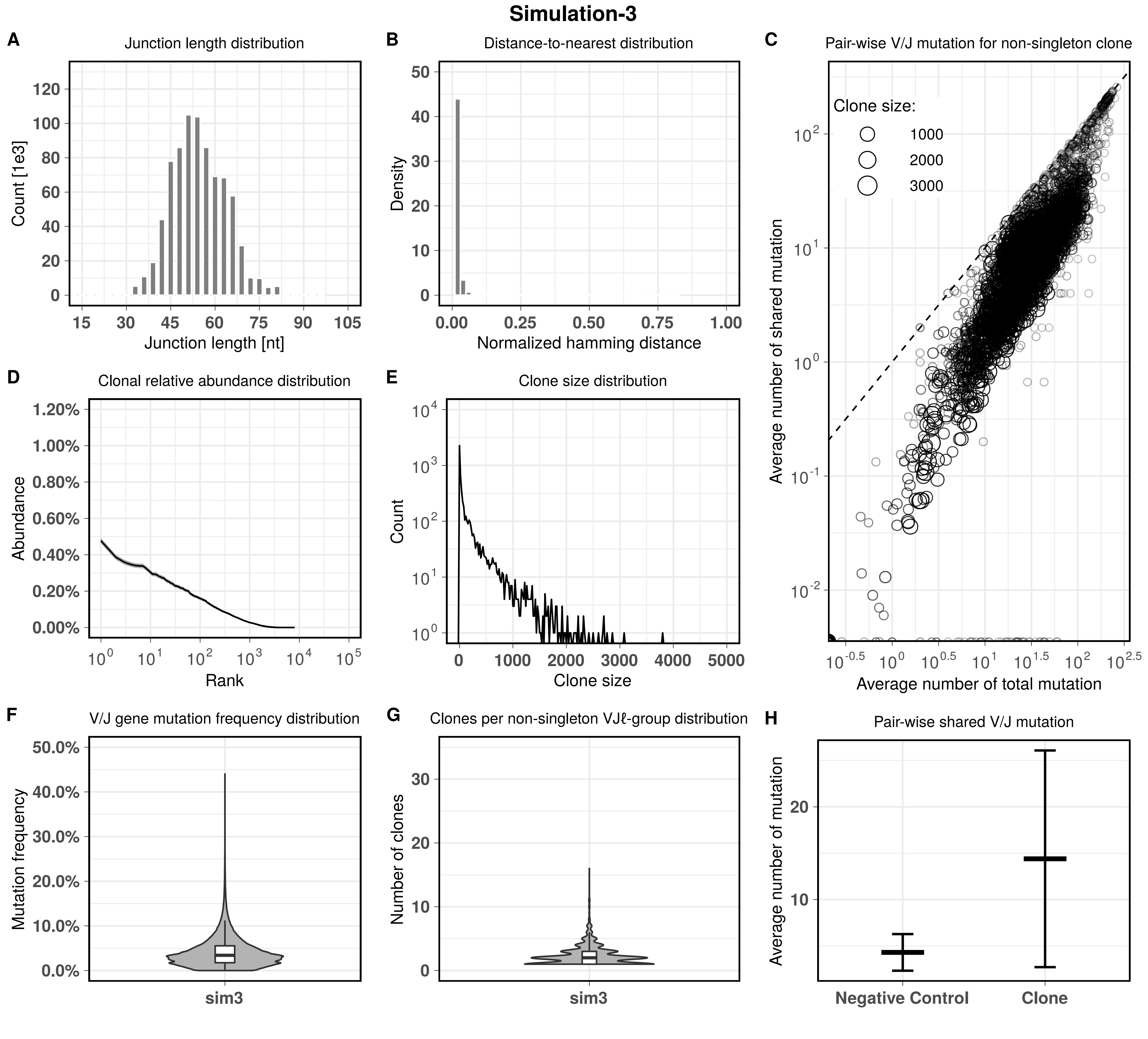

Simulation-3
A
Junction length distribution
Count [1e3]
Junction length [nt]
B
Distance-to-nearest distribution
Density
Normalized hamming distance
C
Pair-wise V/J mutation for non-singleton clone
Clone size:
1000
2000
3000
Average number of shared mutation
Average number of total mutation
D
Clonal relative abundance distribution
Abundance
Rank
E
Clone size distribution
Count
Clone size
F
V/J gene mutation frequency distribution
Mutation frequency
sim3
G
Clones per non-singleton VJℓ-group distribution
Number of clones
sim3
H
Pair-wise shared V/J mutation
Average number of mutation
Negative Control
Clone

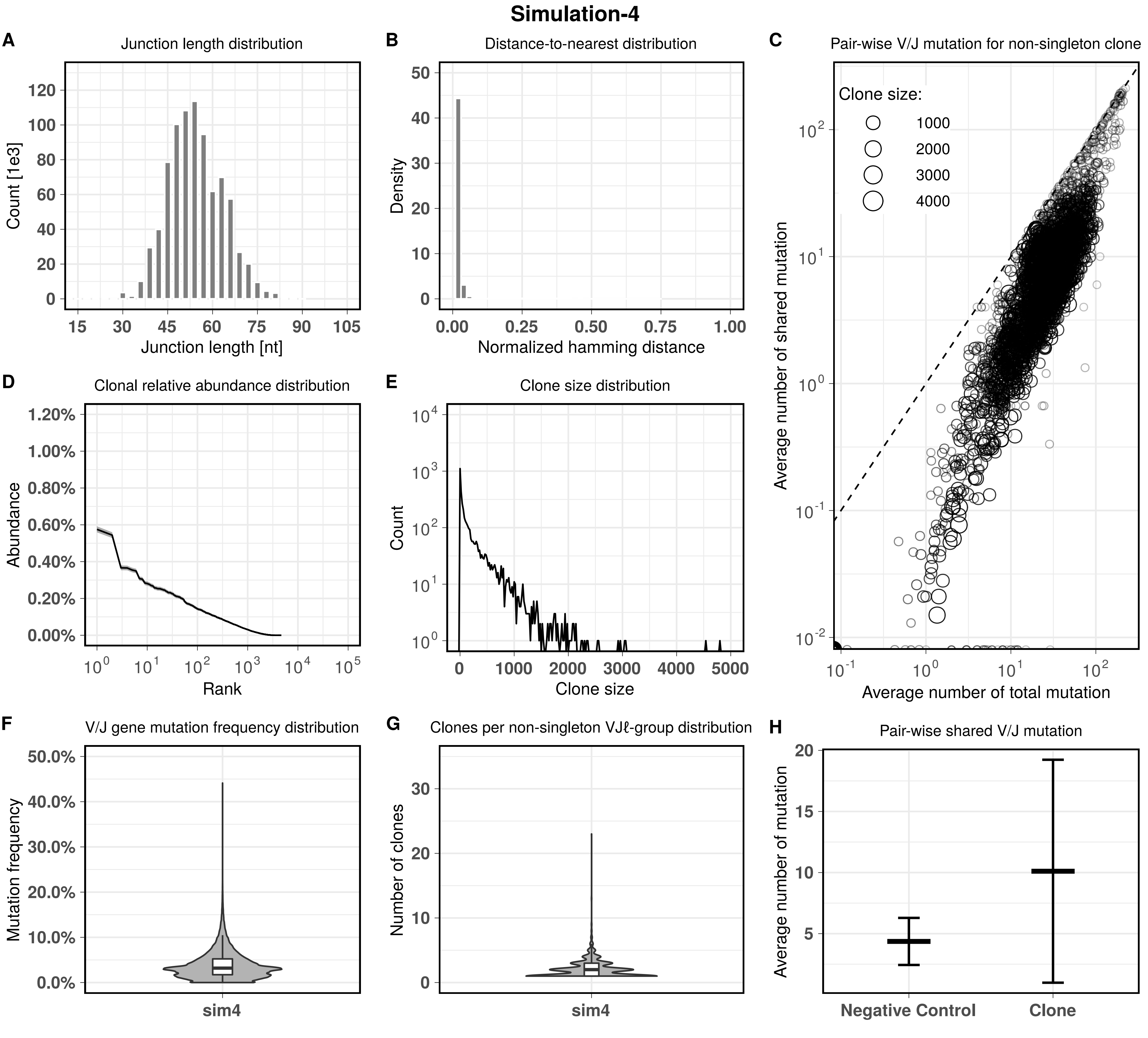
Simulation-4
A
Junction length distribution
Count [1e3]
Junction length [nt]
B
Distance-to-nearest distribution
Density
Normalized hamming distance
C
Pair-wise V/J mutation for non-singleton clone
Clone size:
1000
2000
3000
4000
Average number of shared mutation
Average number of total mutation
D
Clonal relative abundance distribution
Abundance
Rank
E
Clone size distribution
Count
Clone size
F
V/J gene mutation frequency distribution
Mutation frequency
sim4
G
Clones per non-singleton VJℓ-group distribution
Number of clones
sim4
H
Pair-wise shared V/J mutation
Average number of mutation
Negative Control
Clone

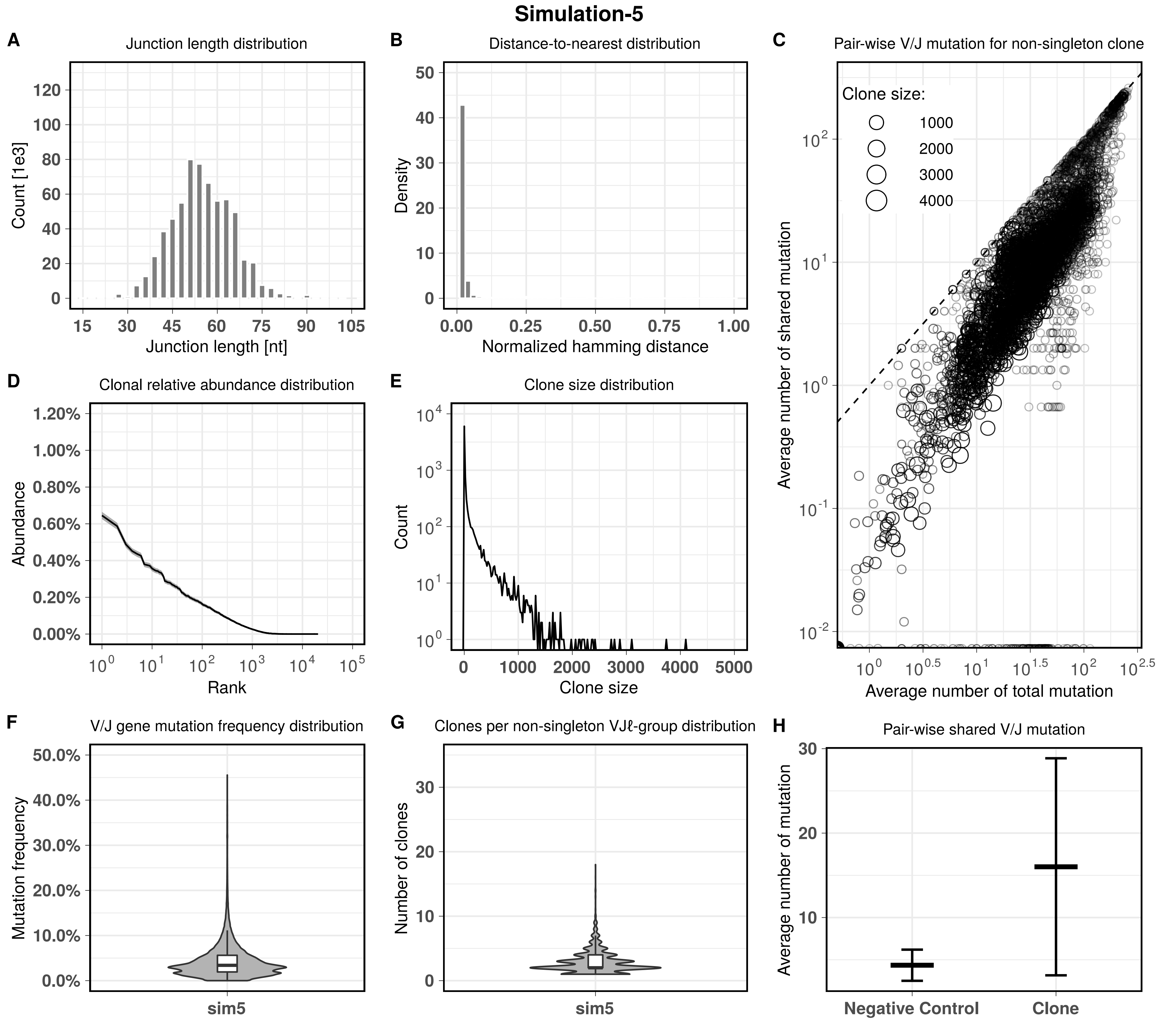

Simulation-5
A
Junction length distribution
Count [1e3]
Junction length [nt]
B
Distance-to-nearest distribution
Density
Normalized hamming distance
C
Pair-wise V/J mutation for non-singleton clone
Clone size:
1000
2000
3000
4000
Average number of shared mutation
Average number of total mutation
D
Clonal relative abundance distribution
Abundance
Rank
E
Clone size distribution
Count
Clone size
F
V/J gene mutation frequency distribution
Mutation frequency
sim5
G
Clones per non-singleton VJℓ-group distribution
Number of clones
sim5
H
Pair-wise shared V/J mutation
Average number of mutation
Negative Control
Clone

Simulation-6

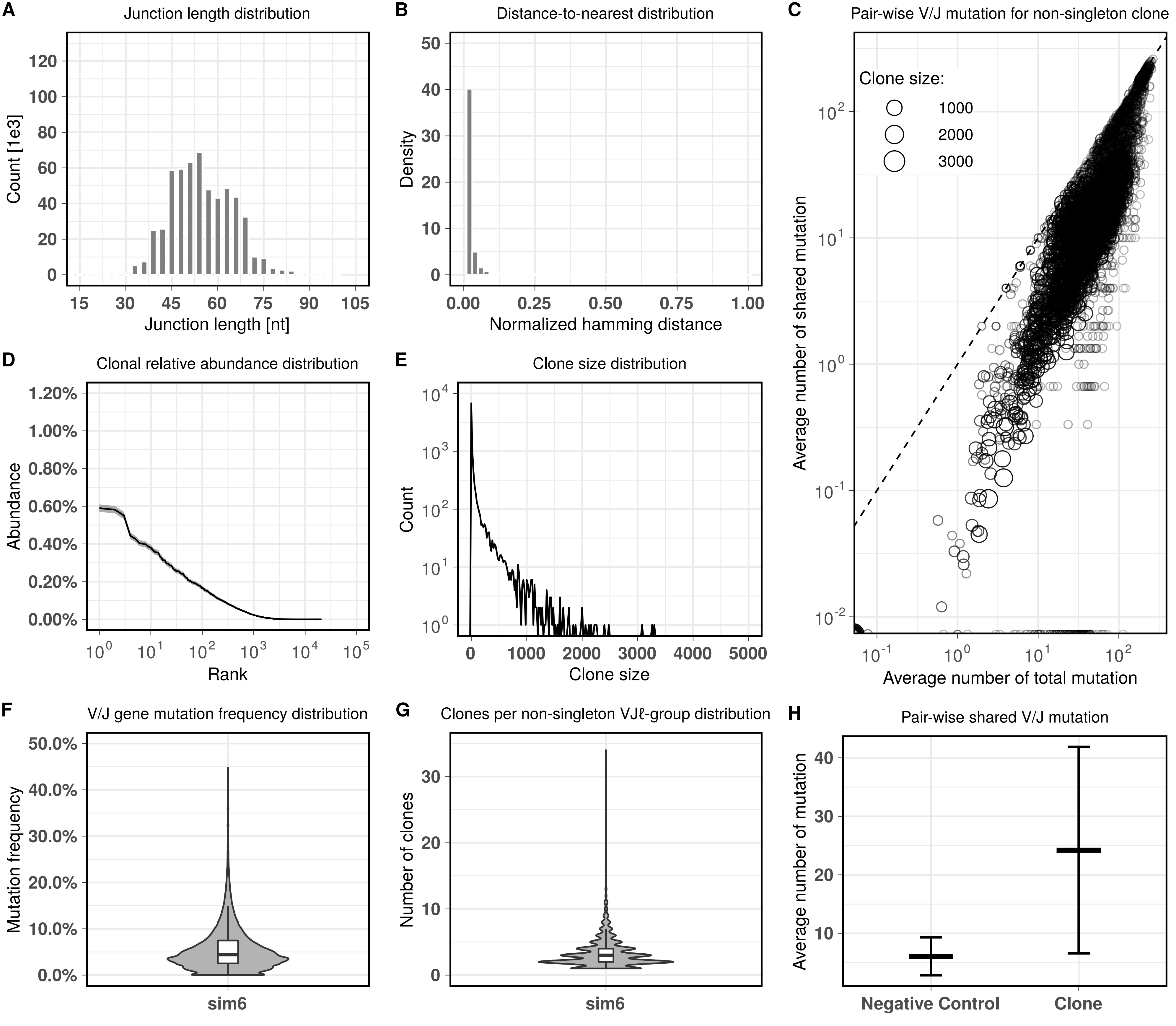

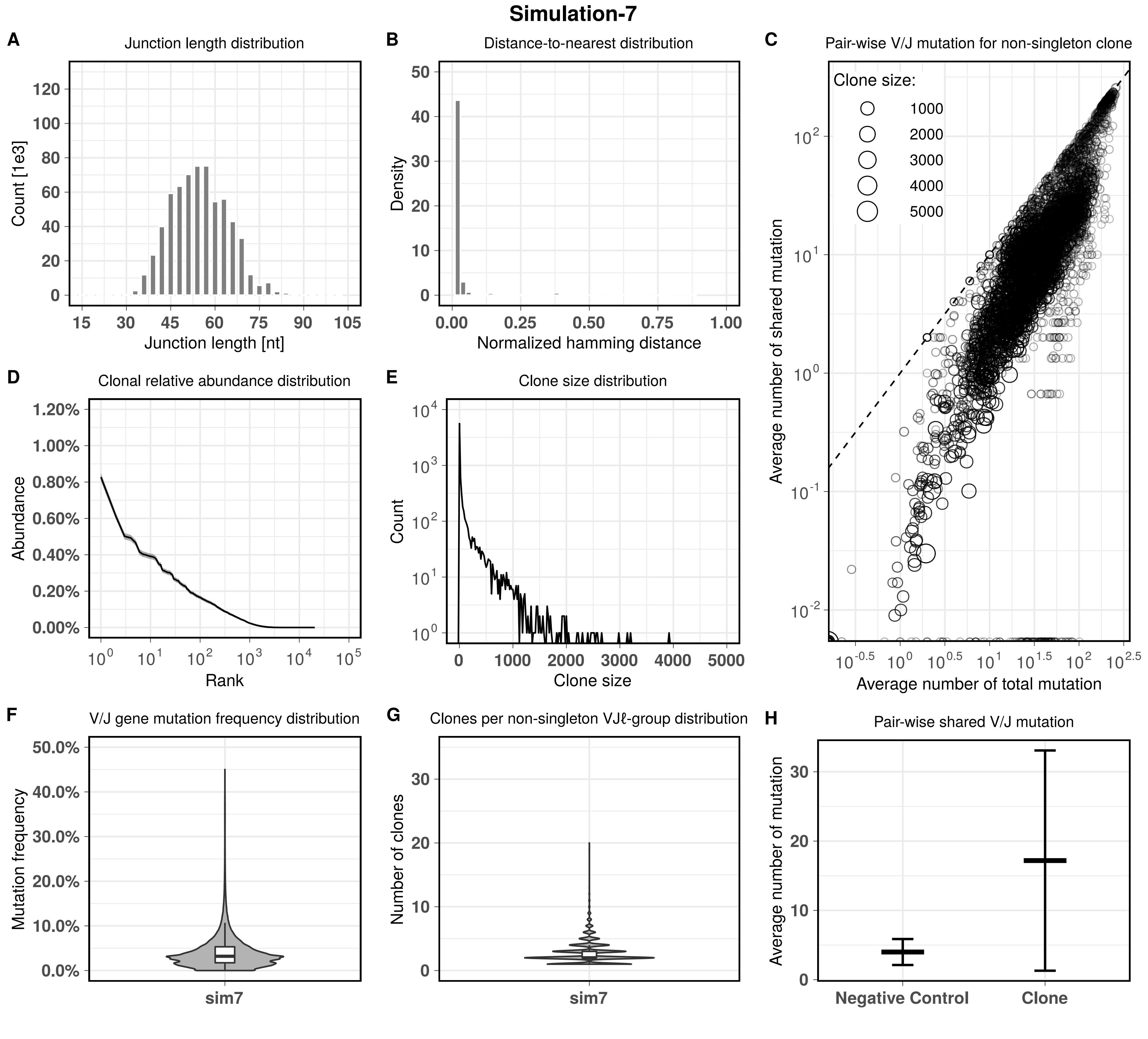
Simulation-7
A
Junction length distribution
Count [1e3]
Junction length [nt]
B
Distance-to-nearest distribution
Density
Normalized hamming distance
C
Pair-wise V/J mutation for non-singleton clone
Clone size:
1000
2000
3000
4000
5000
Average number of shared mutation
Average number of total mutation
D
Clonal relative abundance distribution
Abundance
Rank
E
Clone size distribution
Count
Clone size
F
V/J gene mutation frequency distribution
Mutation frequency
sim7
G
Clones per non-singleton VJℓ-group distribution
Number of clones
sim7
H
Pair-wise shared V/J mutation
Average number of mutation
Negative Control
Clone

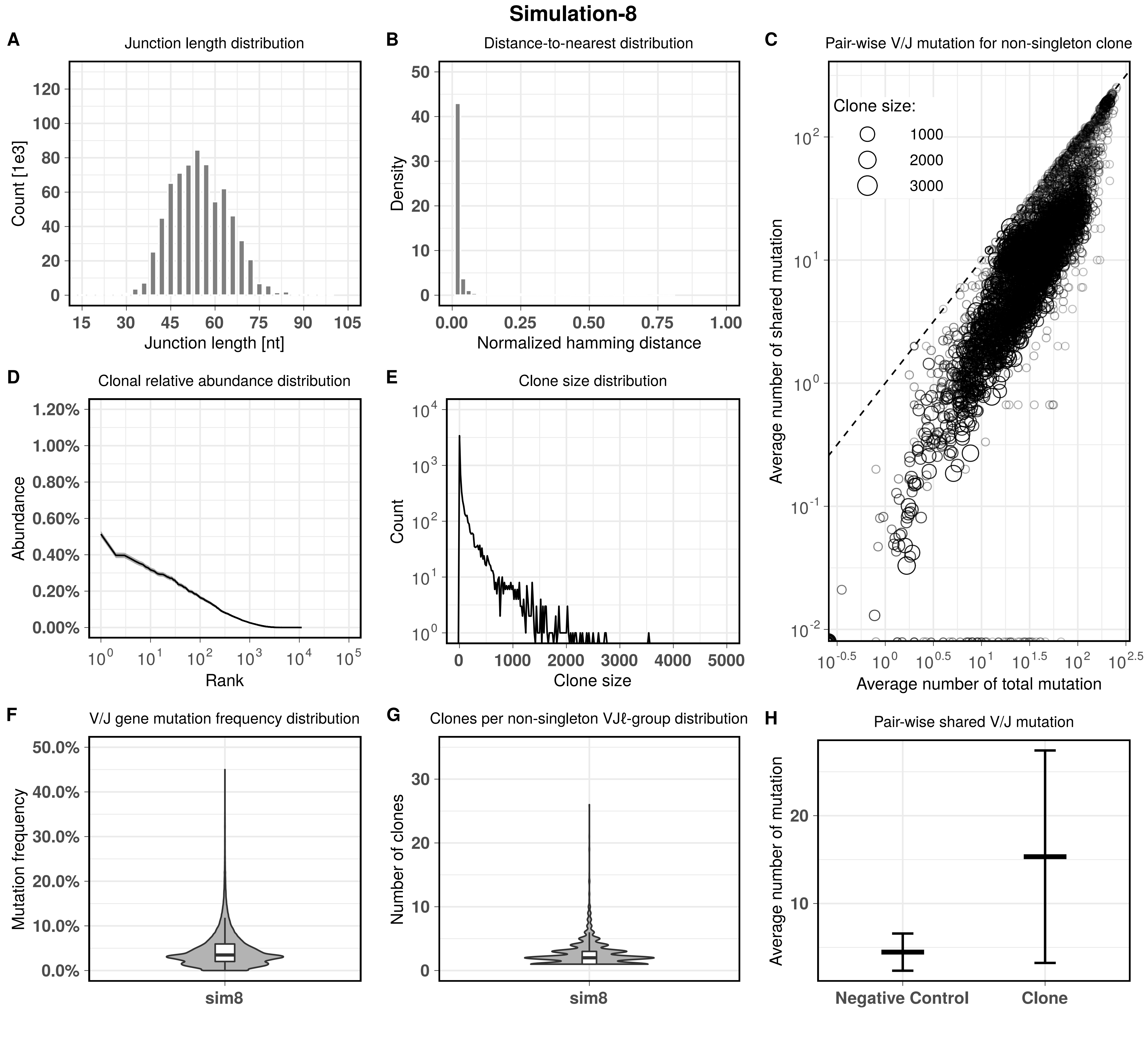

Simulation-8
A
Junction length distribution
Count [1e3]
Junction length [nt]
B
Distance-to-nearest distribution
Density
Normalized hamming distance
C
Pair-wise V/J mutation for non-singleton clone
Clone size:
1000
2000
3000
Average number of shared mutation
Average number of total mutation
D
Clonal relative abundance distribution
Abundance
Rank
E
Clone size distribution
Count
Clone size
F
V/J gene mutation frequency distribution
Mutation frequency
sim8
G
Clones per non-singleton VJℓ-group distribution
Number of clones
sim8
H
Pair-wise shared V/J mutation
Average number of mutation
Negative Control
Clone

Simulation-9

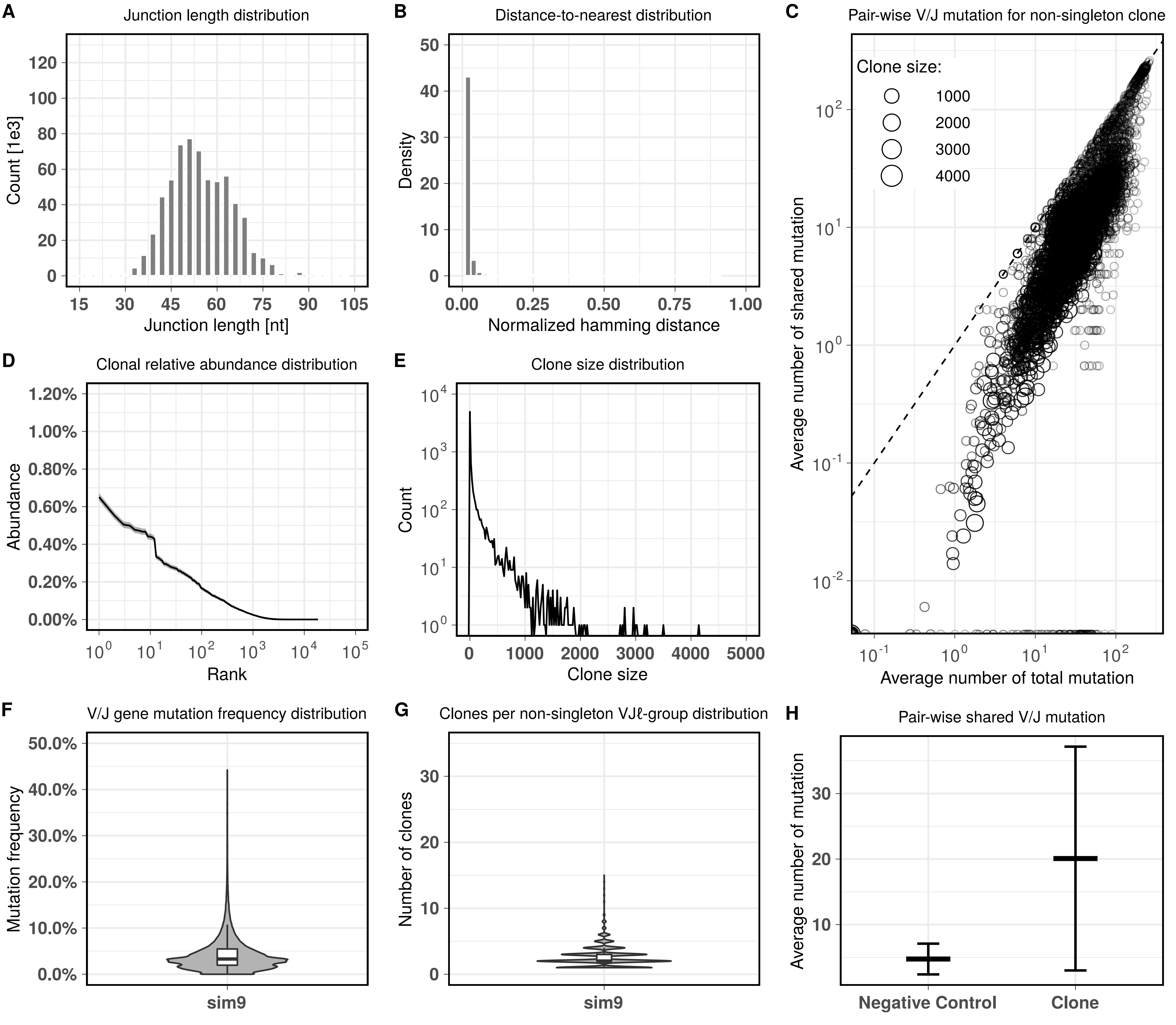

Simulation-10

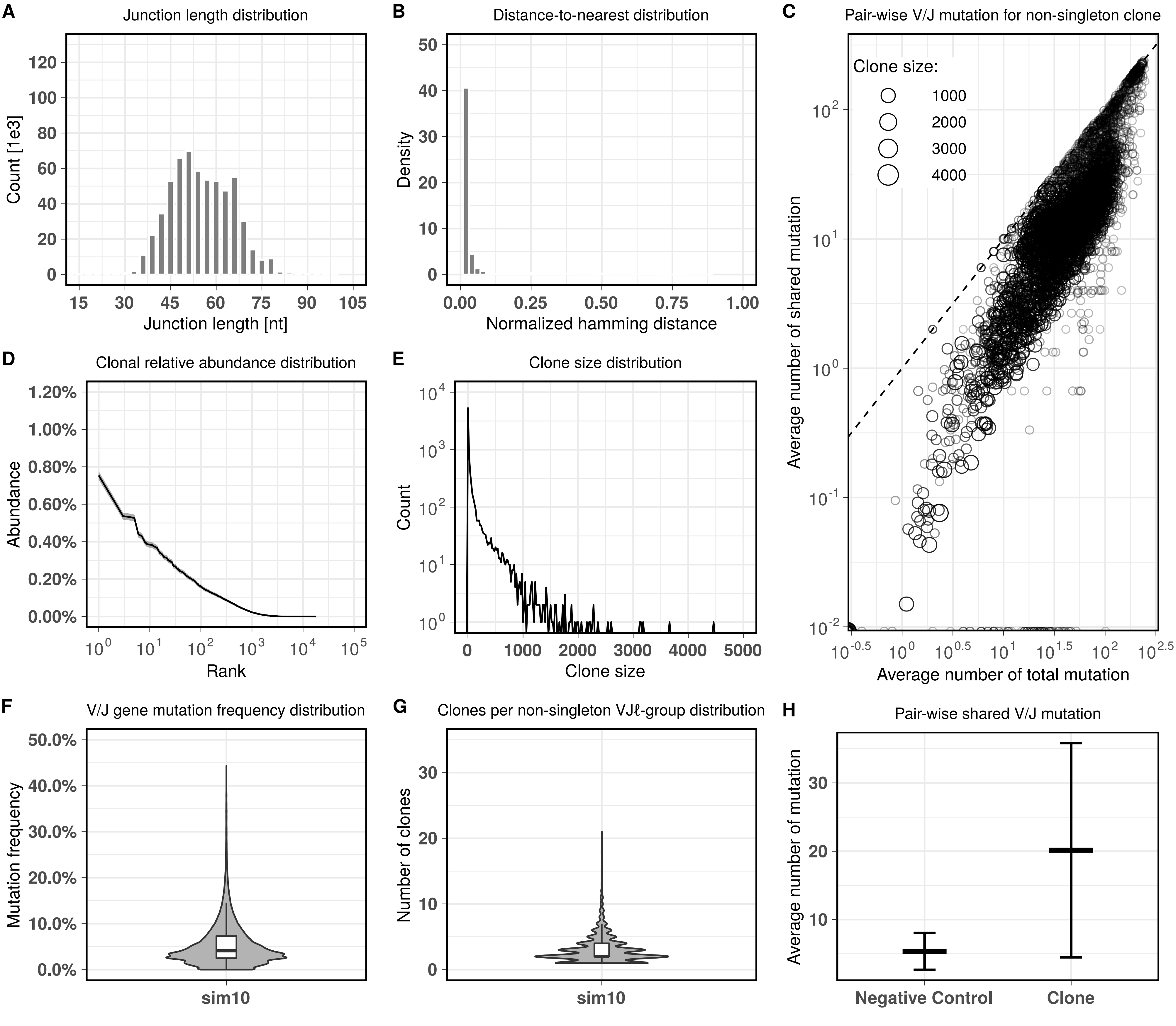

Simulation-11

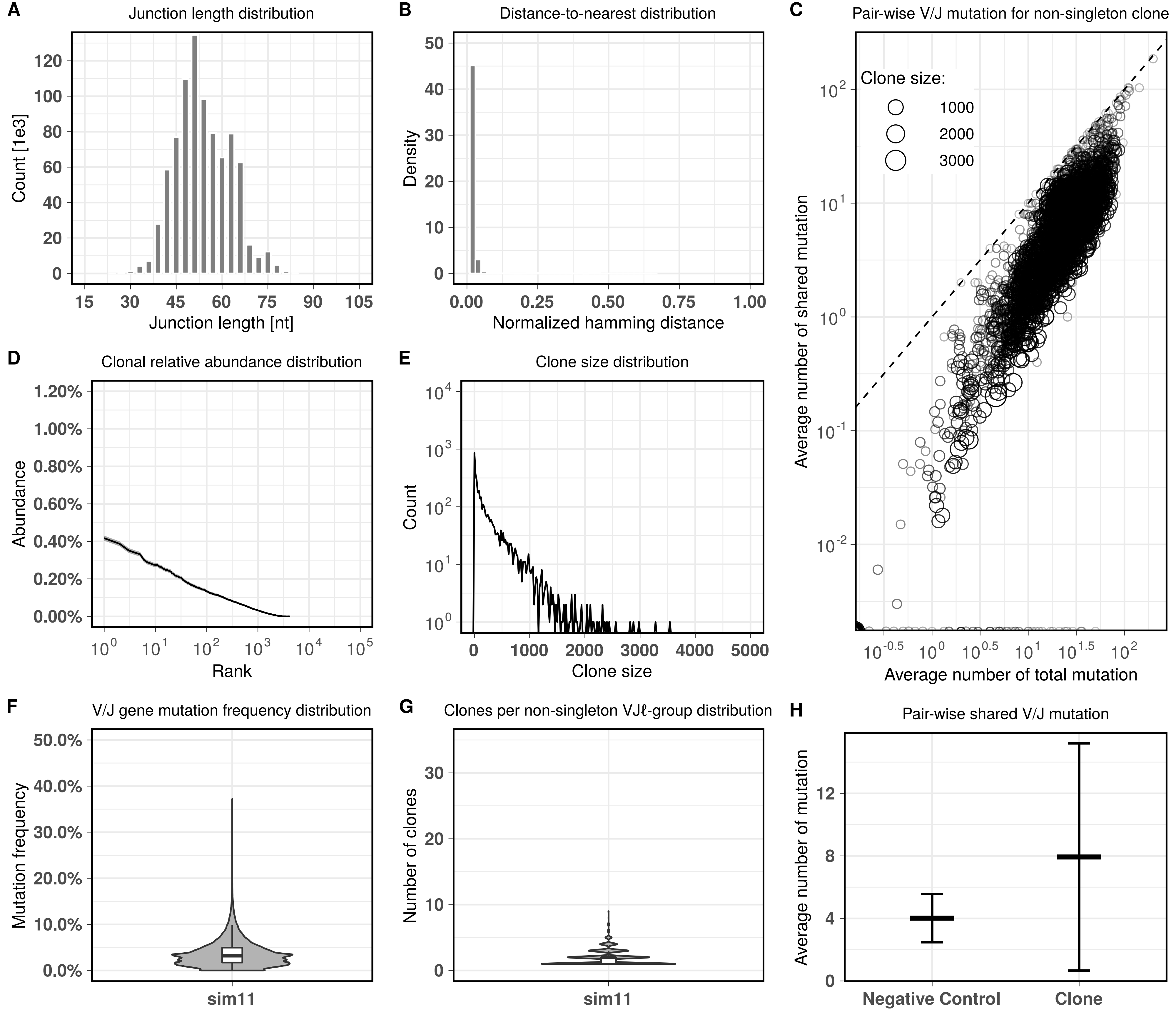

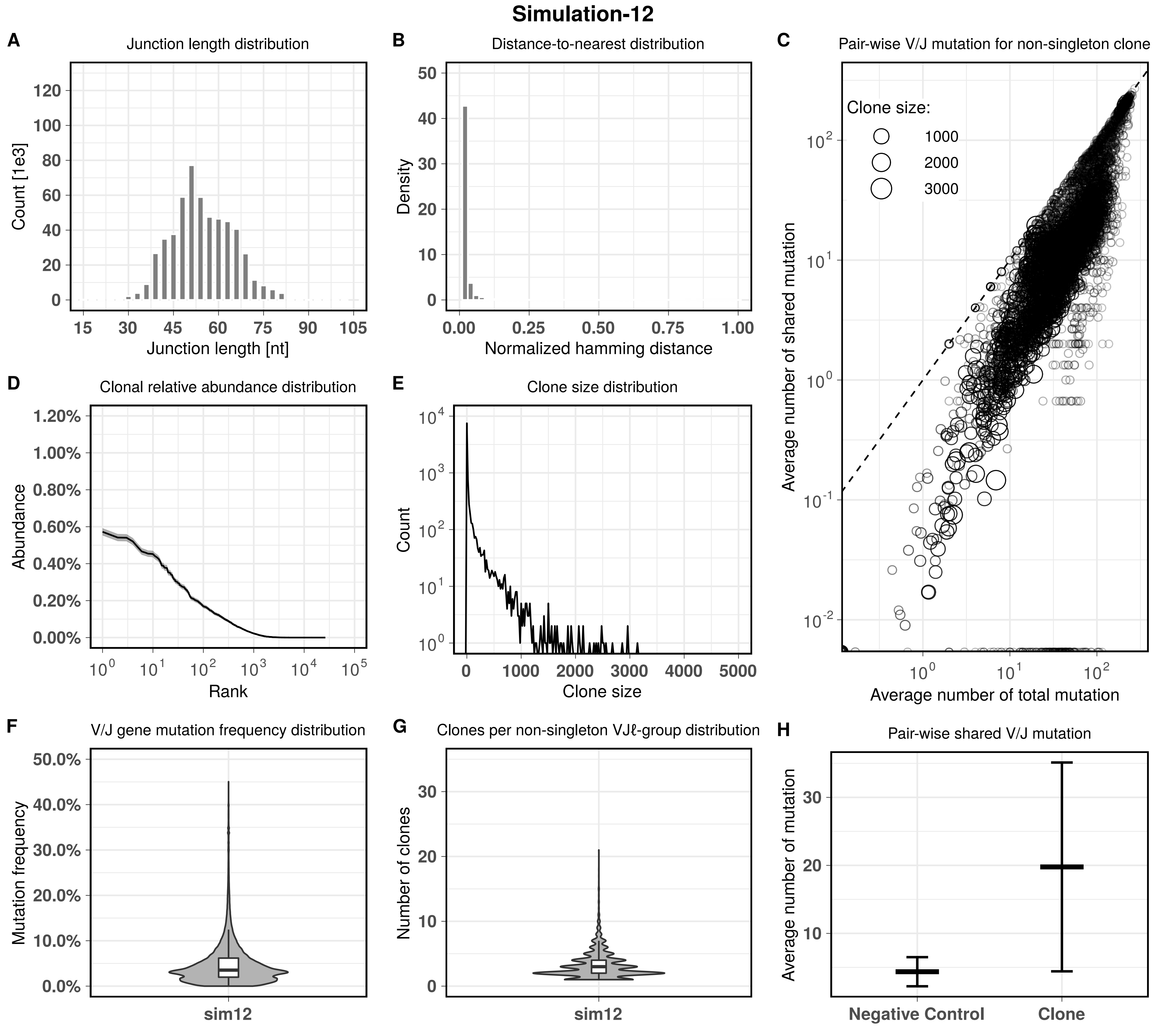
Simulation-12
A
Junction length distribution
Count [1e3]
Junction length [nt]
B
Distance-to-nearest distribution
Density
Normalized hamming distance
C
Pair-wise V/J mutation for non-singleton clone
Clone size:
1000
2000
3000
Average number of shared mutation
Average number of total mutation
D
Clonal relative abundance distribution
Abundance
Rank
E
Clone size distribution
Count
Clone size
F
V/J gene mutation frequency distribution
Mutation frequency
sim12
G
Clones per non-singleton VJℓ-group distribution
Number of clones
sim12
H
Pair-wise shared V/J mutation
Average number of mutation
Negative Control
Clone

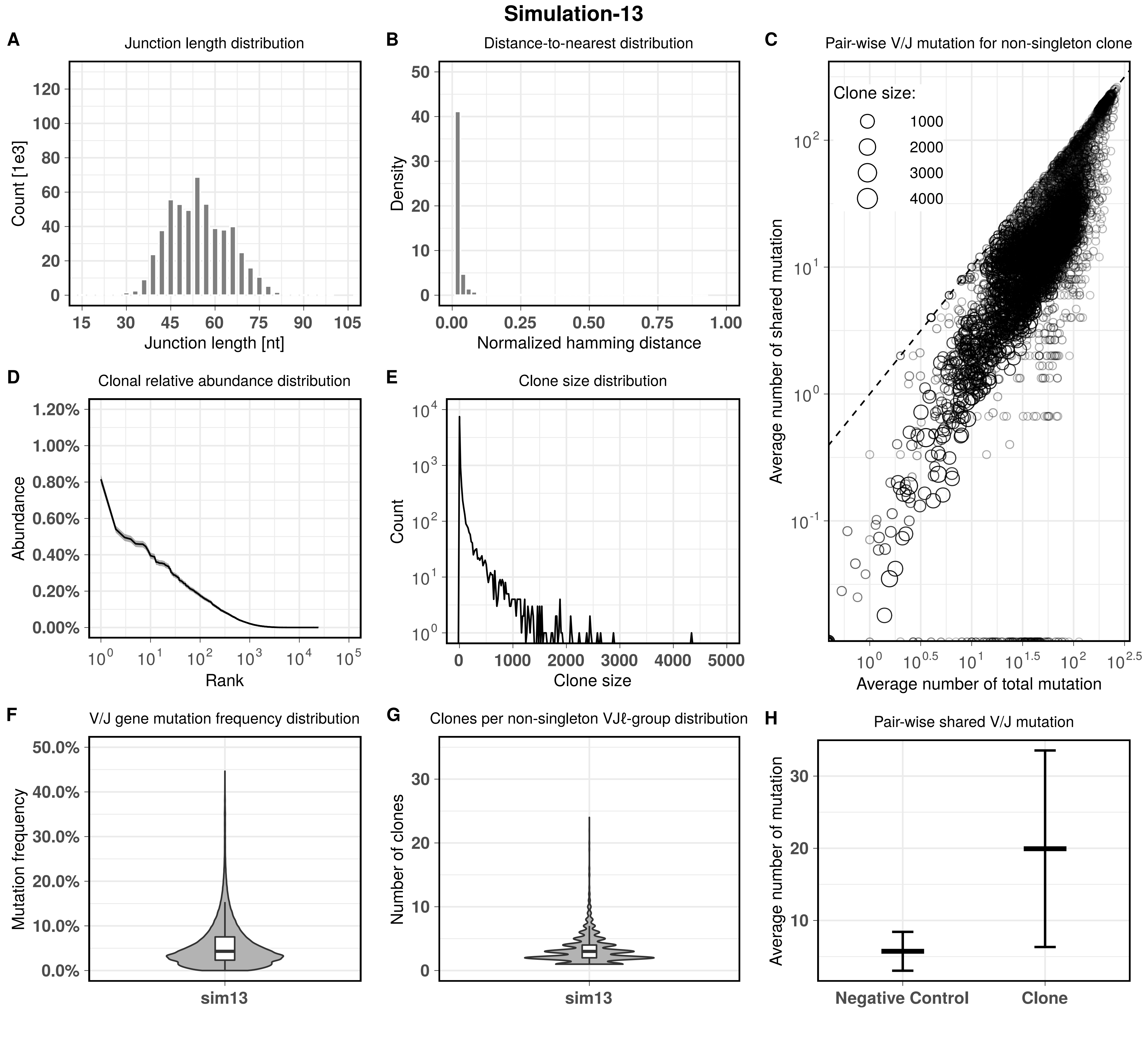
Simulation-13
A
Junction length distribution
Count [1e3]
Junction length [nt]
B
Distance-to-nearest distribution
Density
Normalized hamming distance
C
Pair-wise V/J mutation for non-singleton clone
Clone size:
1000
2000
3000
4000
Average number of shared mutation
Average number of total mutation
D
Clonal relative abundance distribution
Abundance
Rank
E
Clone size distribution
Count
Clone size
F
V/J gene mutation frequency distribution
Mutation frequency
sim13
G
Clones per non-singleton VJℓ-group distribution
Number of clones
sim13
H
Pair-wise shared V/J mutation
Average number of mutation
Negative Control
Clone

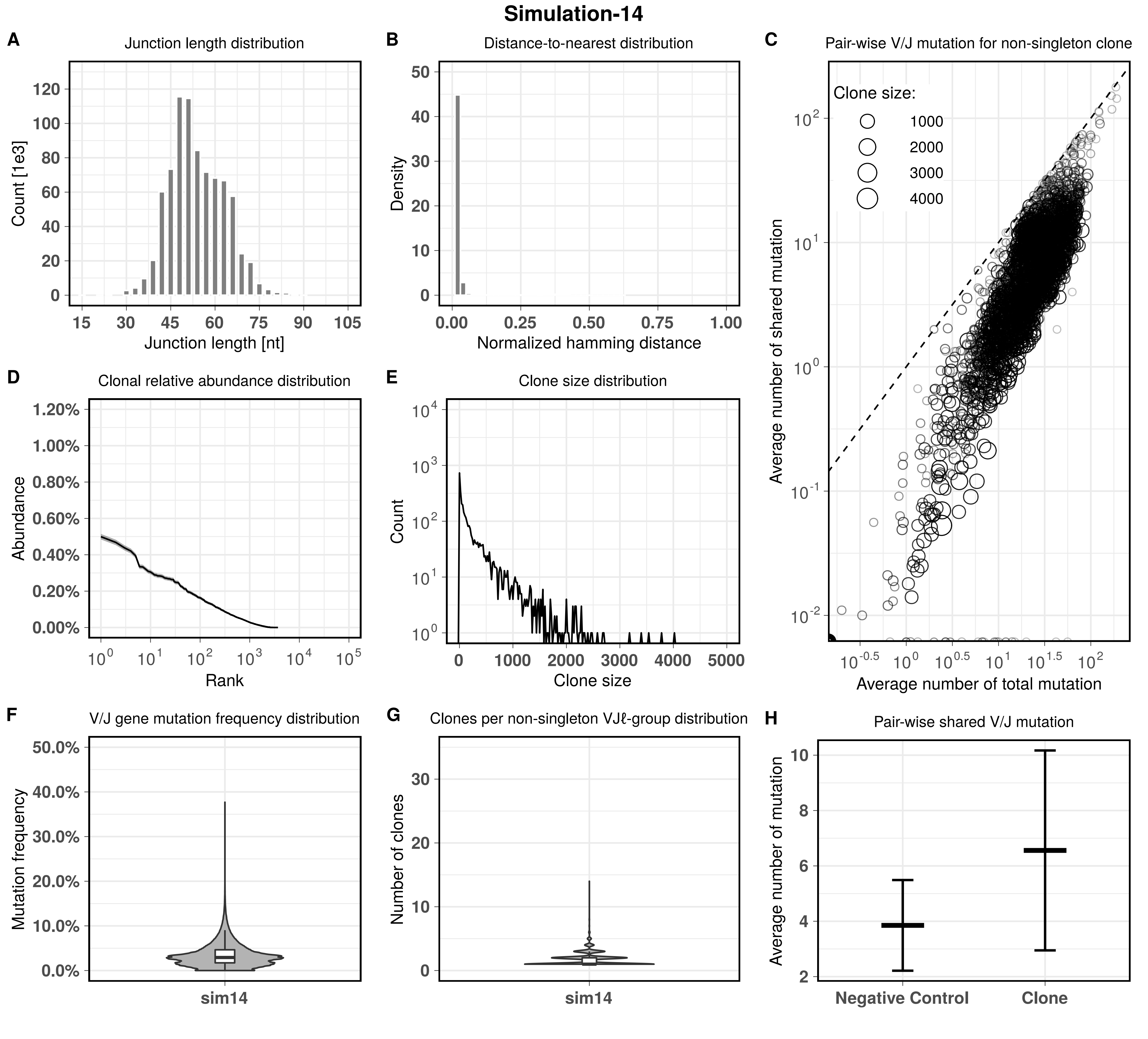
Simulation-14
A
Junction length distribution
Count [1e3]
Junction length [nt]
B
Distance-to-nearest distribution
Density
Normalized hamming distance
C
Pair-wise V/J mutation for non-singleton clone
Clone size:
1000
2000
3000
4000
Average number of shared mutation
Average number of total mutation
D
Clonal relative abundance distribution
Abundance
Rank
E
Clone size distribution
Count
Clone size
F
V/J gene mutation frequency distribution
Mutation frequency
sim14
G
Clones per non-singleton VJℓ-group distribution
Number of clones
sim14
H
Pair-wise shared V/J mutation
Average number of mutation
Negative Control
Clone

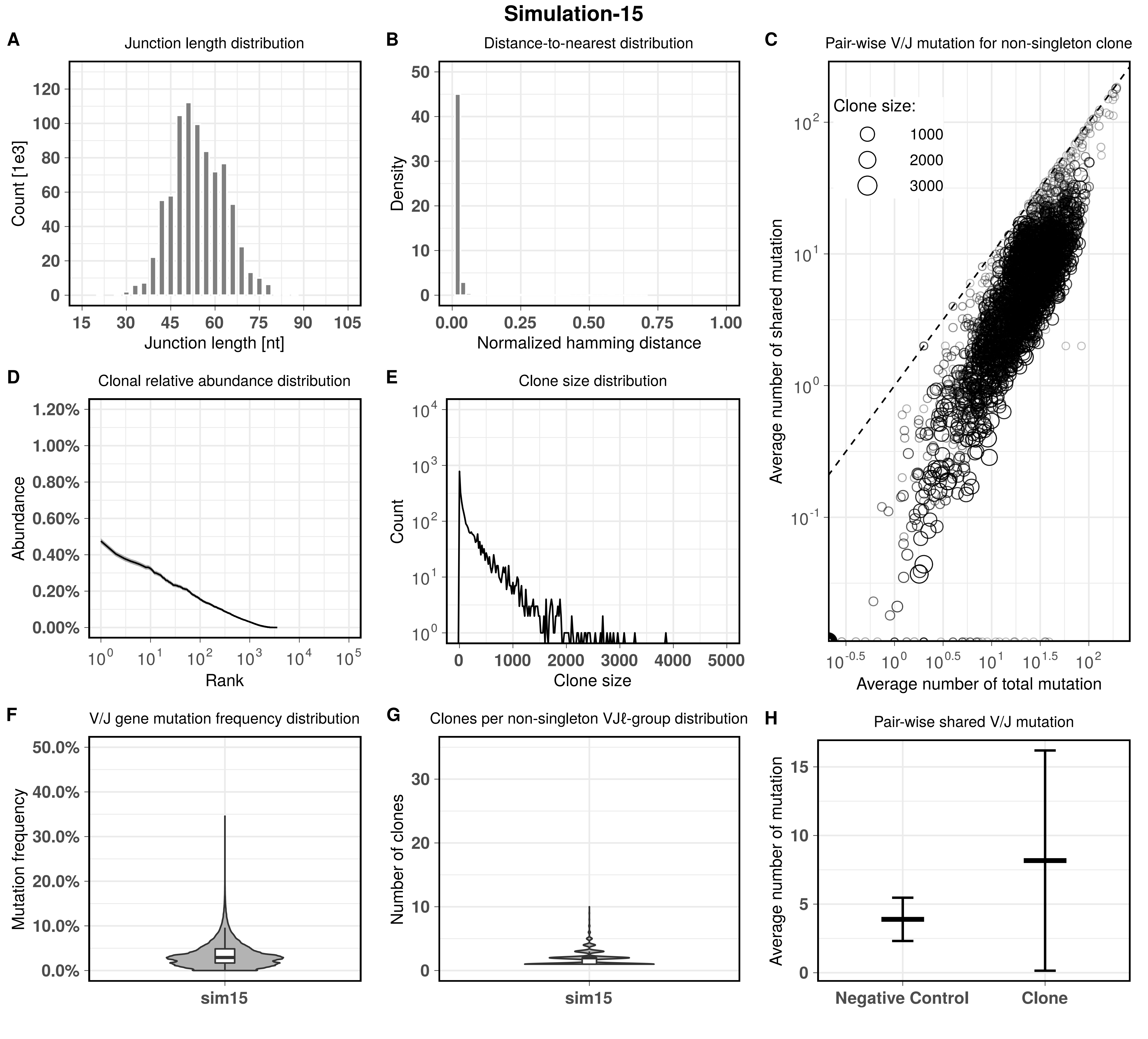

Simulation-15
A
Junction length distribution
Count [1e3]
Junction length [nt]
B
Distance-to-nearest distribution
Density
Normalized hamming distance
C
Pair-wise V/J mutation for non-singleton clone
Clone size:
1000
2000
3000
Average number of shared mutation
Average number of total mutation
D
Clonal relative abundance distribution
Abundance
Rank
E
Clone size distribution
Count
Clone size
F
V/J gene mutation frequency distribution
Mutation frequency
sim15
G
Clones per non-singleton VJℓ-group distribution
Number of clones
sim15
H
Pair-wise shared V/J mutation
Average number of mutation
Negative Control
Clone

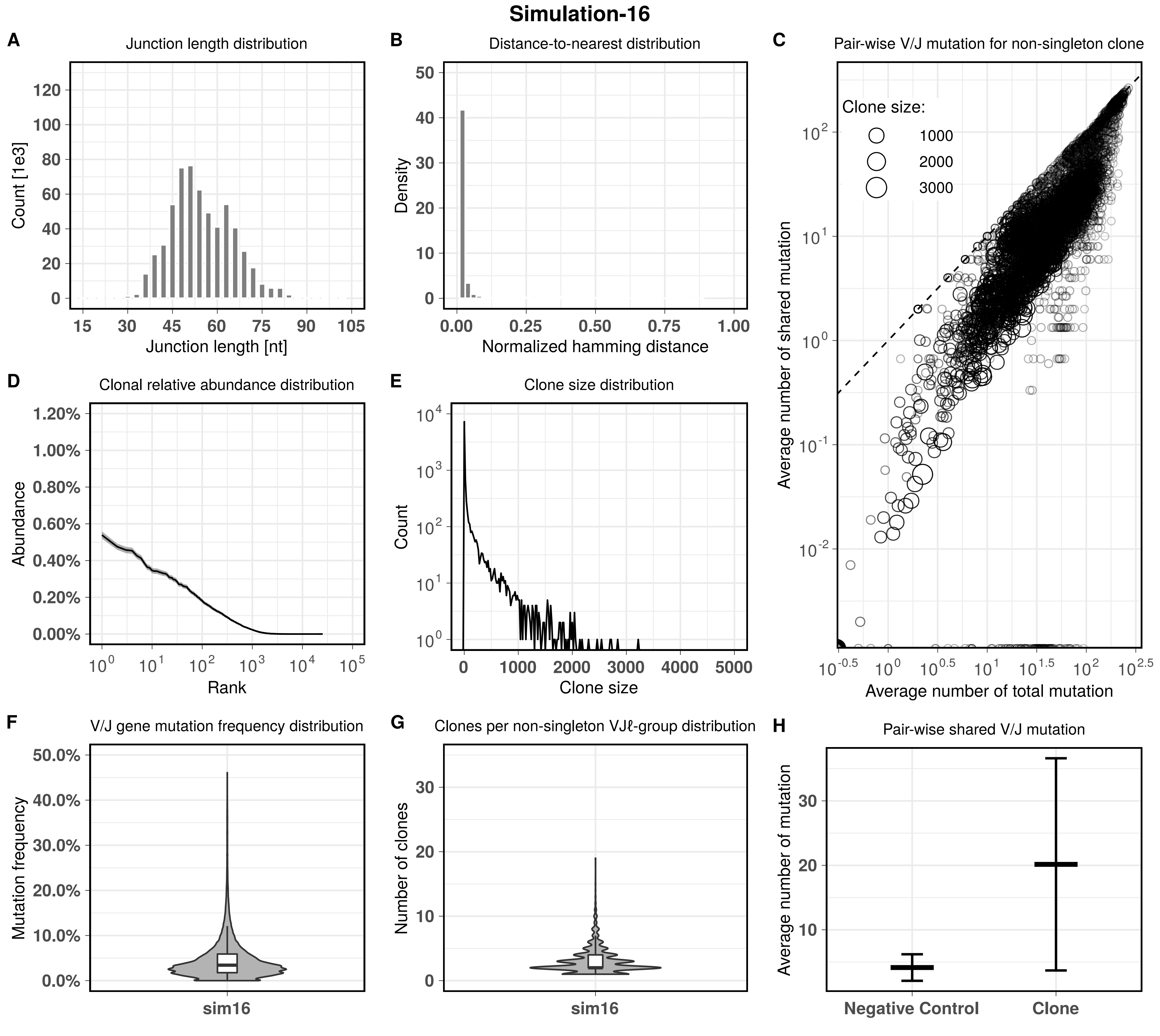
Simulation-16
A
Junction length distribution
Count [1e3]
Junction length [nt]
B
Distance-to-nearest distribution
Density
Normalized hamming distance
C
Pair-wise V/J mutation for non-singleton clone
Clone size:
1000
2000
3000
Average number of shared mutation
Average number of total mutation
D
Clonal relative abundance distribution
Abundance
Rank
E
Clone size distribution
Count
Clone size
F
V/J gene mutation frequency distribution
Mutation frequency
sim16
G
Clones per non-singleton VJℓ-group distribution
Number of clones
sim16
H
Pair-wise shared V/J mutation
Average number of mutation
Negative Control
Clone

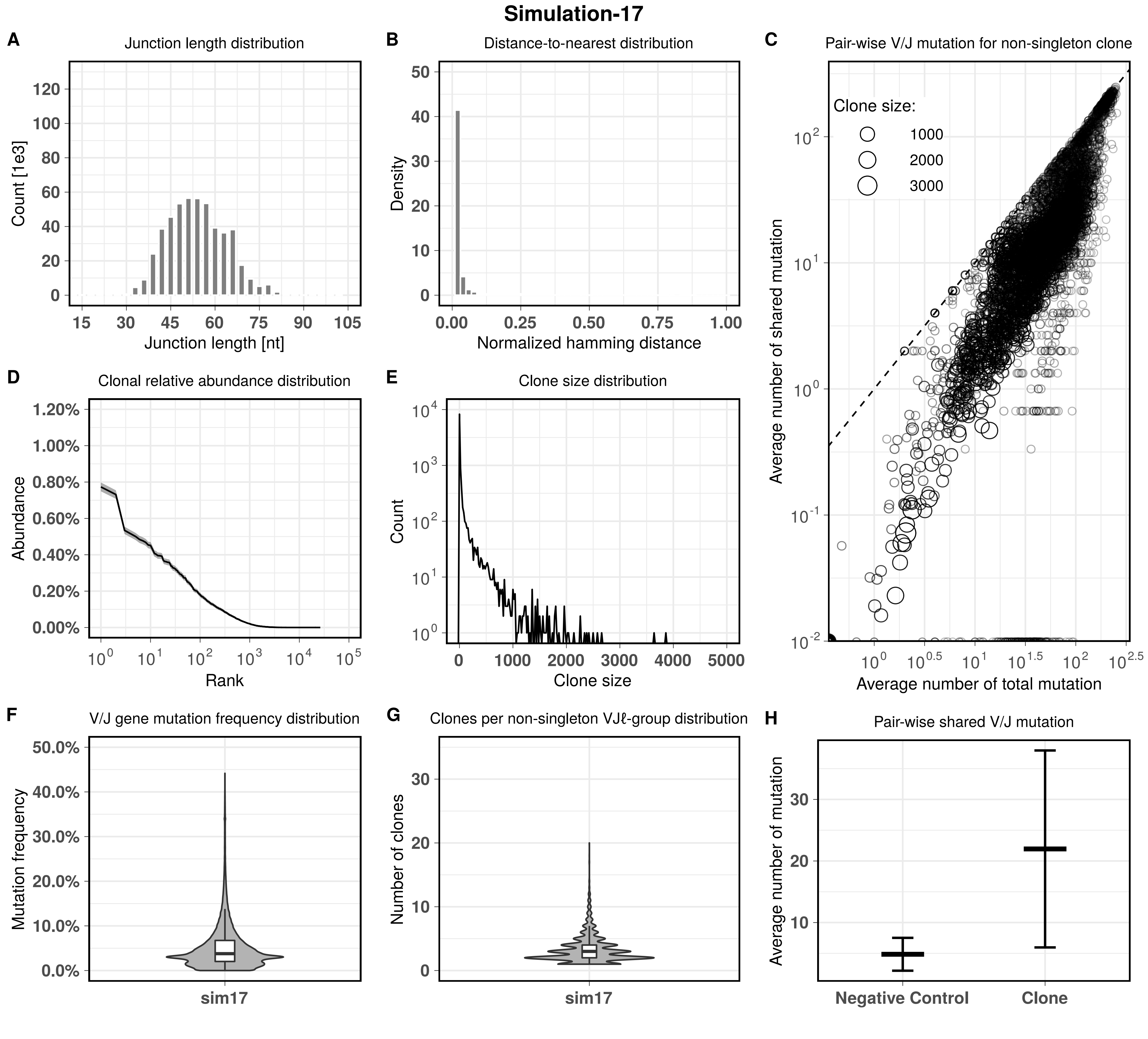
Simulation-17
A
Junction length distribution
Count [1e3]
Junction length [nt]
B
Distance-to-nearest distribution
Density
Normalized hamming distance
C
Pair-wise V/J mutation for non-singleton clone
Clone size:
1000
2000
3000
Average number of shared mutation
Average number of total mutation
D
Clonal relative abundance distribution
Abundance
Rank
E
Clone size distribution
Count
Clone size
F
V/J gene mutation frequency distribution
Mutation frequency
sim17
G
Clones per non-singleton VJℓ-group distribution
Number of clones
sim17
H
Pair-wise shared V/J mutation
Average number of mutation
Negative Control
Clone

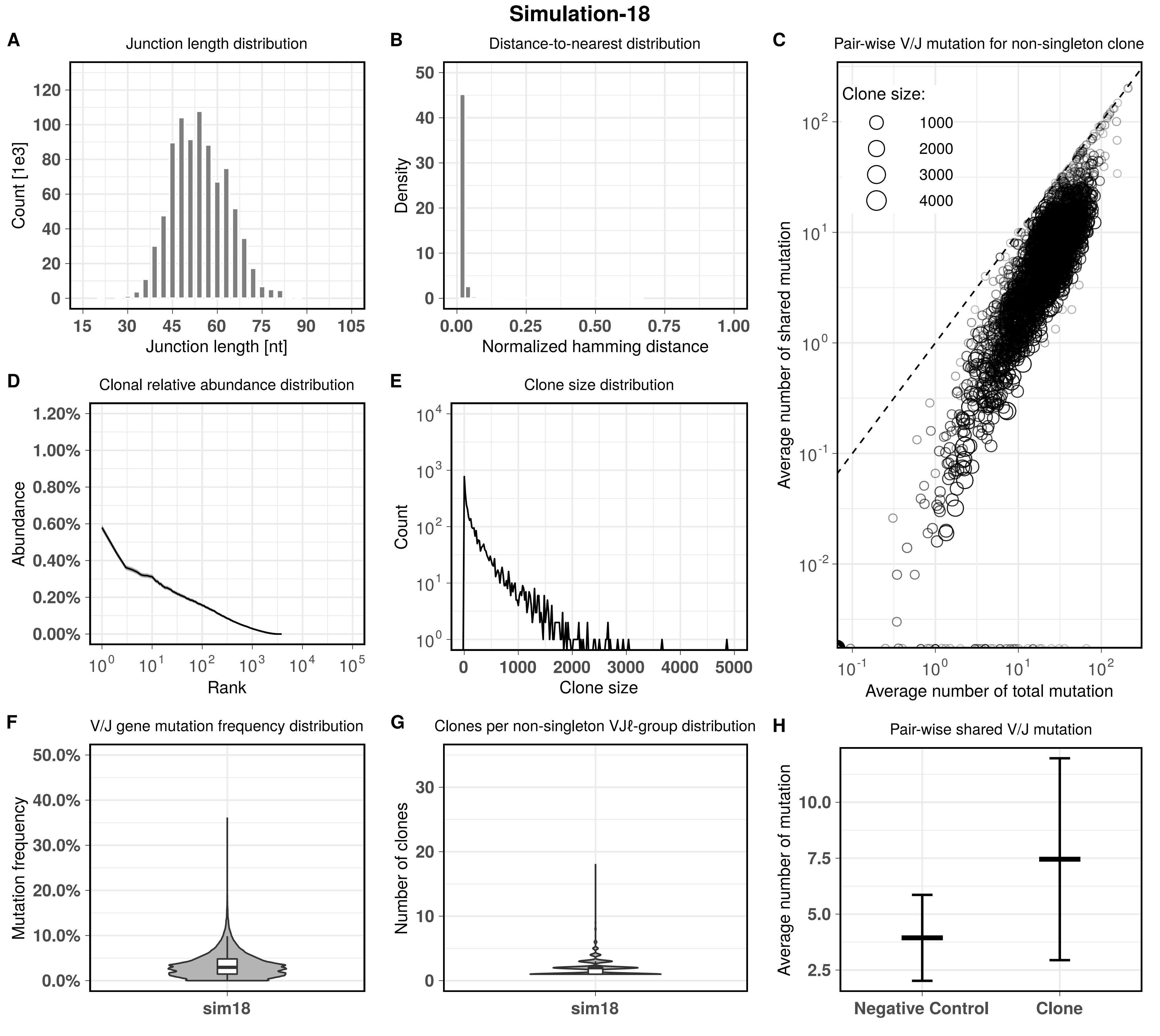
Simulation-18
A
Junction length distribution
Count [1e3]
Junction length [nt]
B
Distance-to-nearest distribution
Density
Normalized hamming distance
C
Pair-wise V/J mutation for non-singleton clone
Clone size:
1000
2000
3000
4000
Average number of shared mutation
Average number of total mutation
D
Clonal relative abundance distribution
Abundance
Rank
E
Clone size distribution
Count
Clone size
F
V/J gene mutation frequency distribution
Mutation frequency
sim18
G
Clones per non-singleton VJℓ-group distribution
Number of clones
sim18
H
Pair-wise shared V/J mutation
Average number of mutation
Negative Control
Clone

Simulation-19

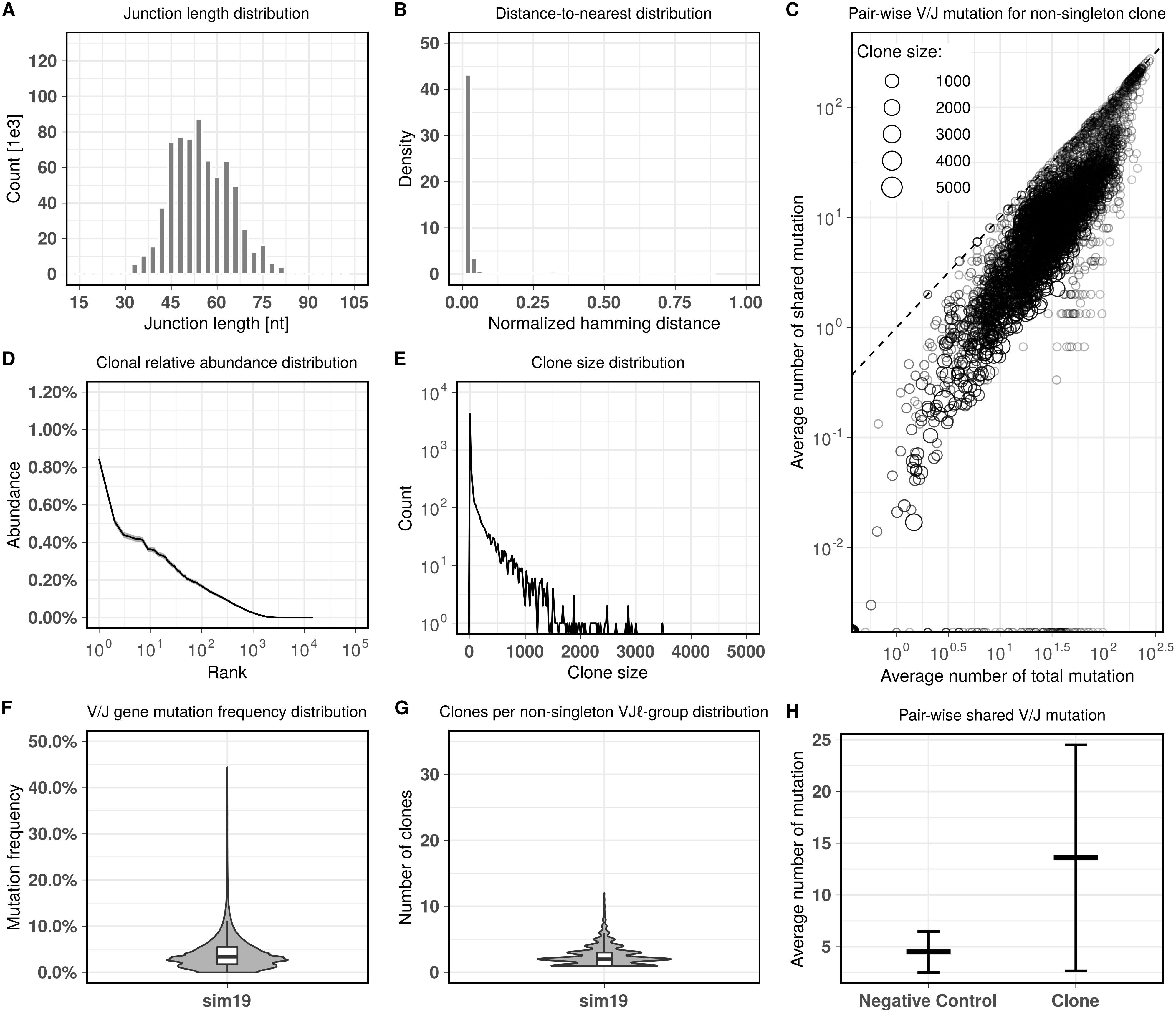

Simulation-20

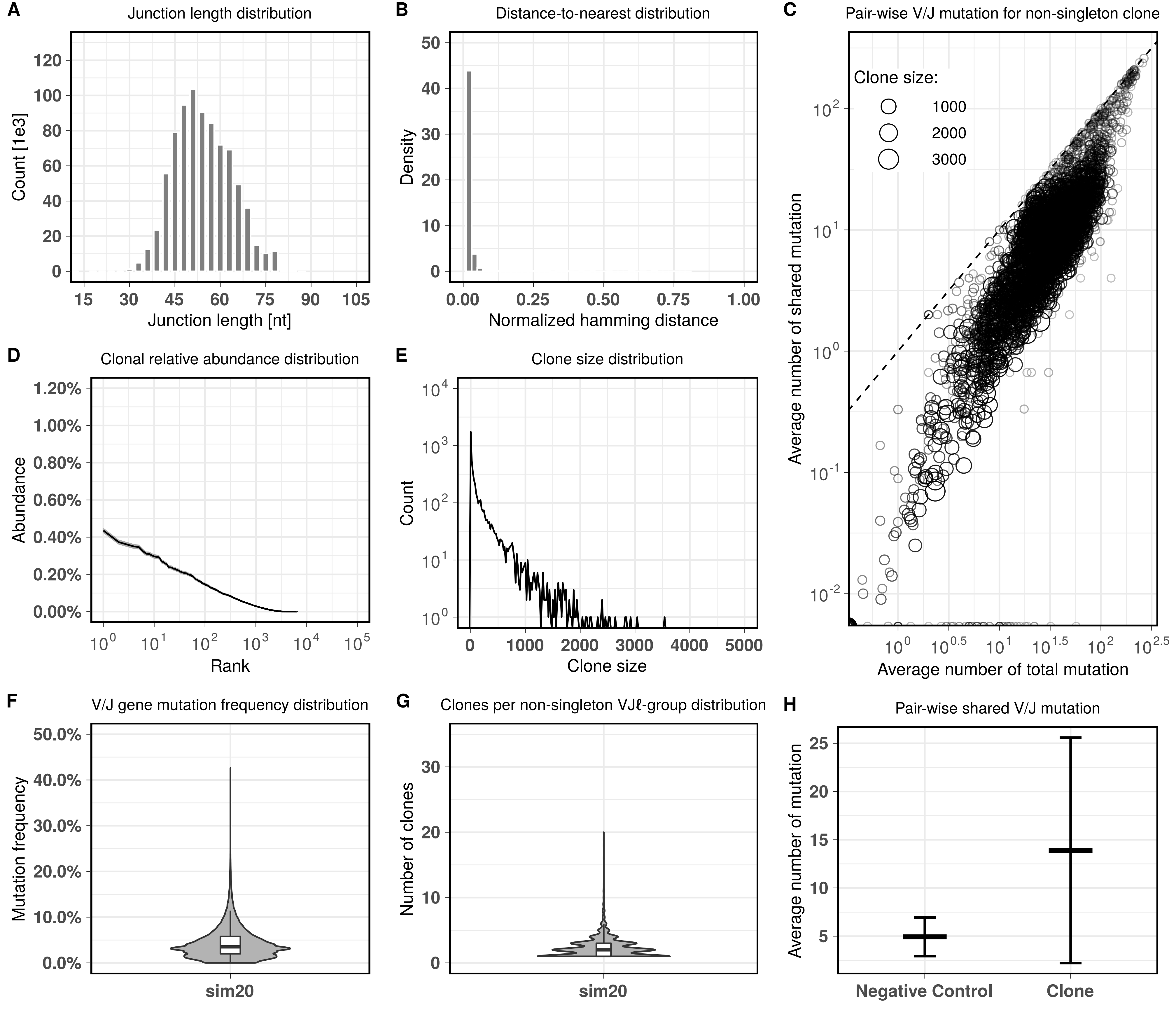

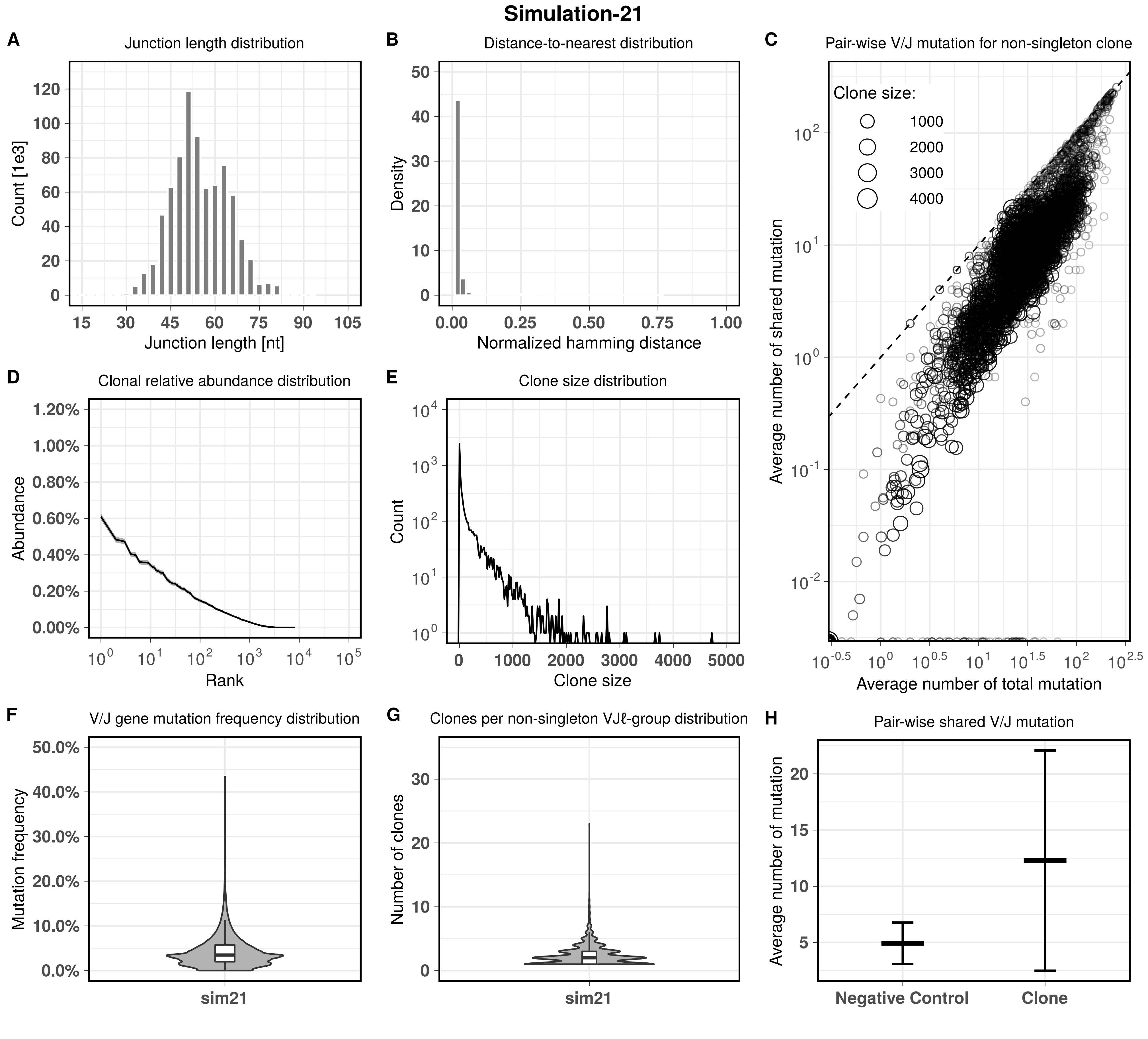

Simulation-21
A
Junction length distribution
Count [1e3]
Junction length [nt]
B
Distance-to-nearest distribution
Density
Normalized hamming distance
C
Pair-wise V/J mutation for non-singleton clone
Clone size:
1000
2000
3000
4000
Average number of shared mutation
Average number of total mutation
D
Clonal relative abundance distribution
Abundance
Rank
E
Clone size distribution
Count
Clone size
F
V/J gene mutation frequency distribution
Mutation frequency
sim21
G
Clones per non-singleton VJℓ-group distribution
Number of clones
sim21
H
Pair-wise shared V/J mutation
Average number of mutation
Negative Control
Clone

Simulation-22

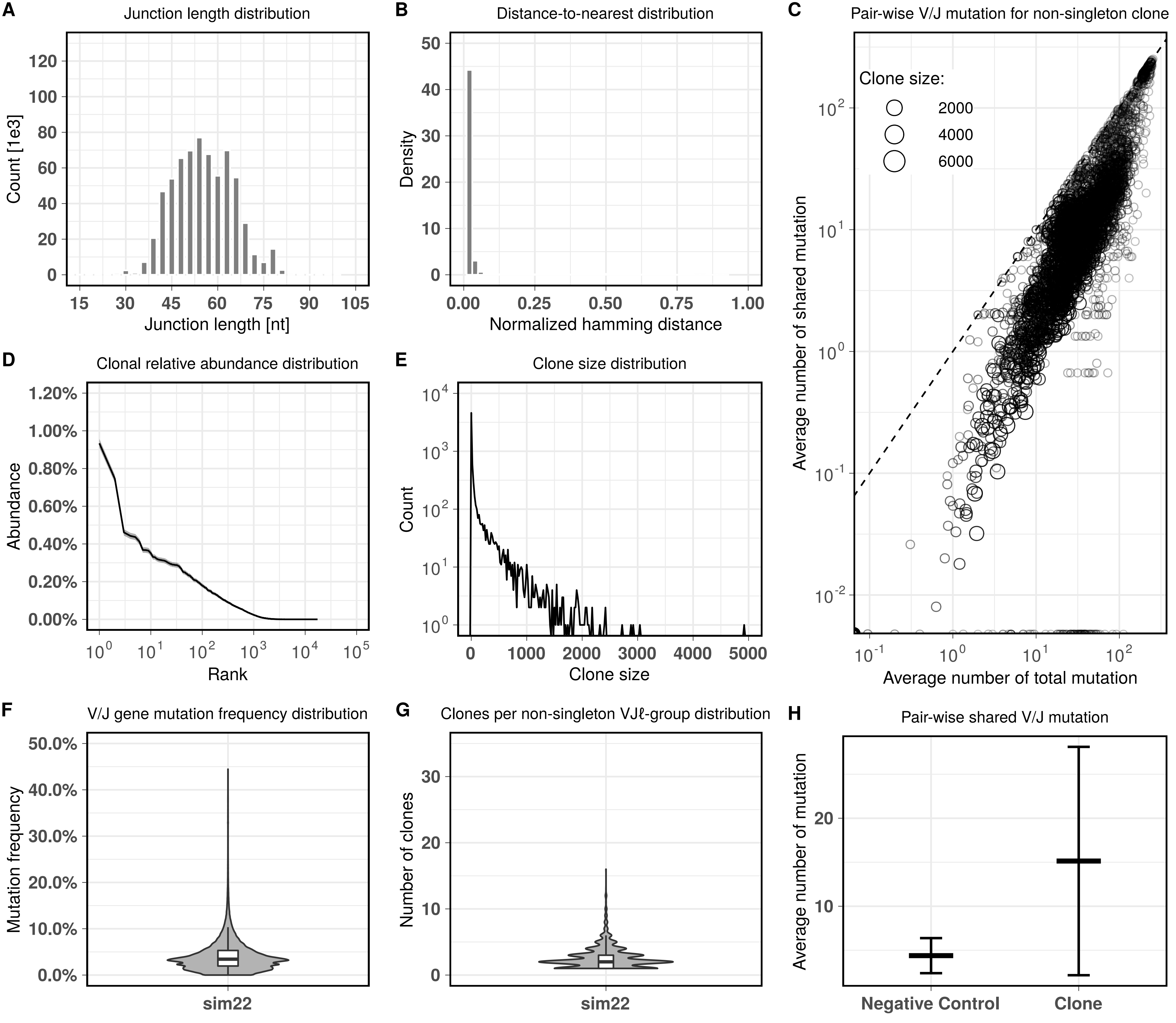

Simulation-23

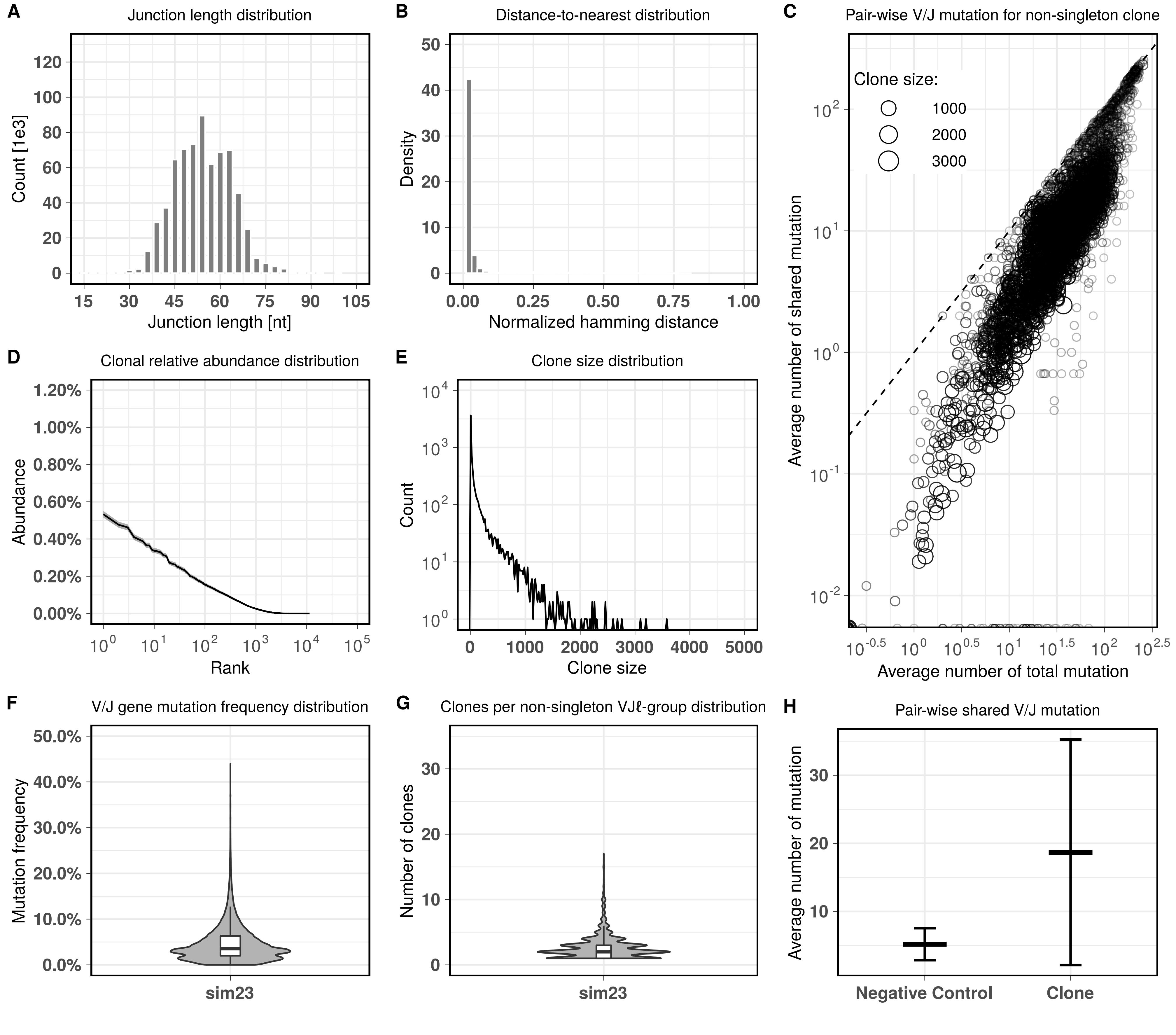

Simulation-24

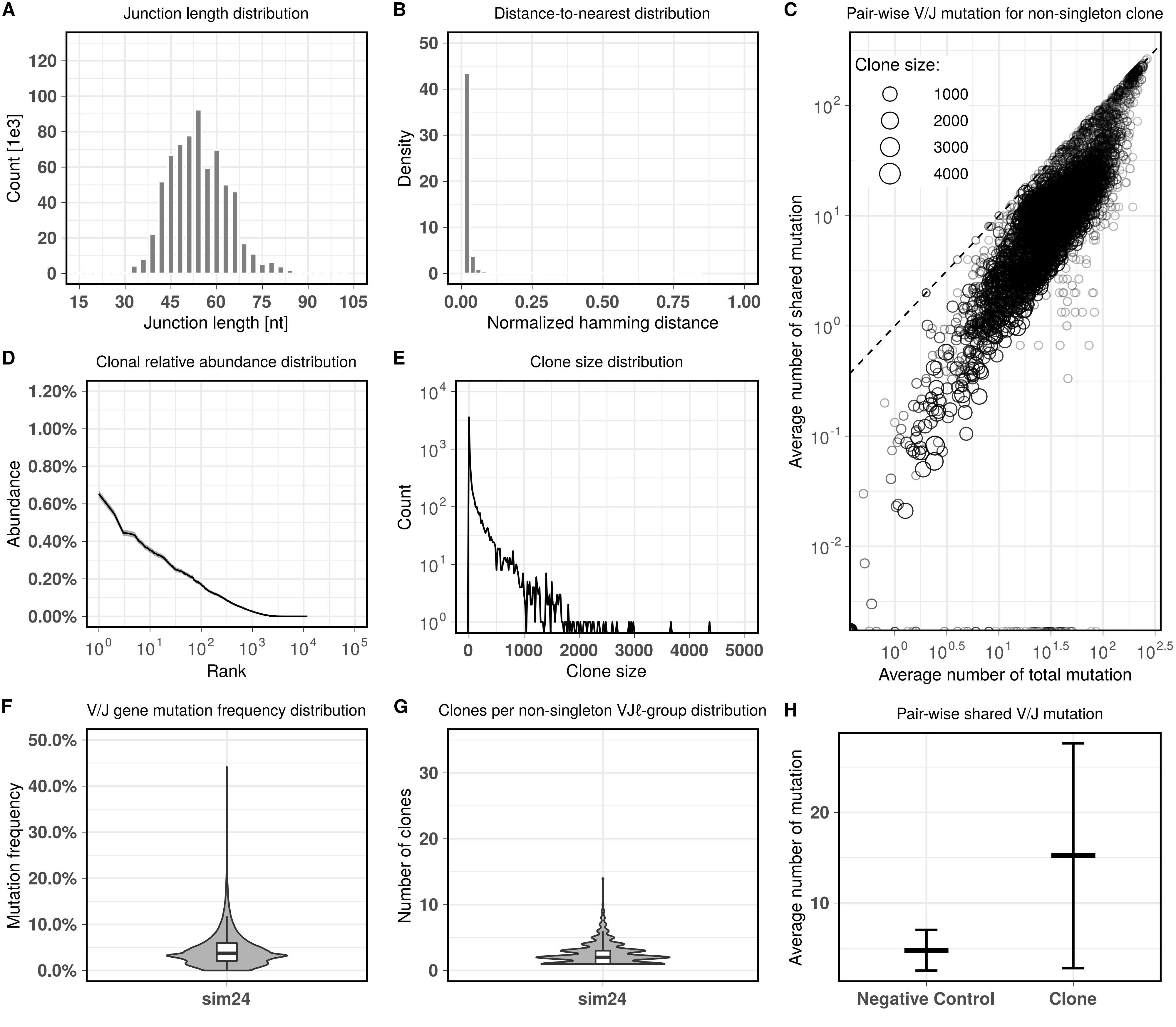

Simulation-25

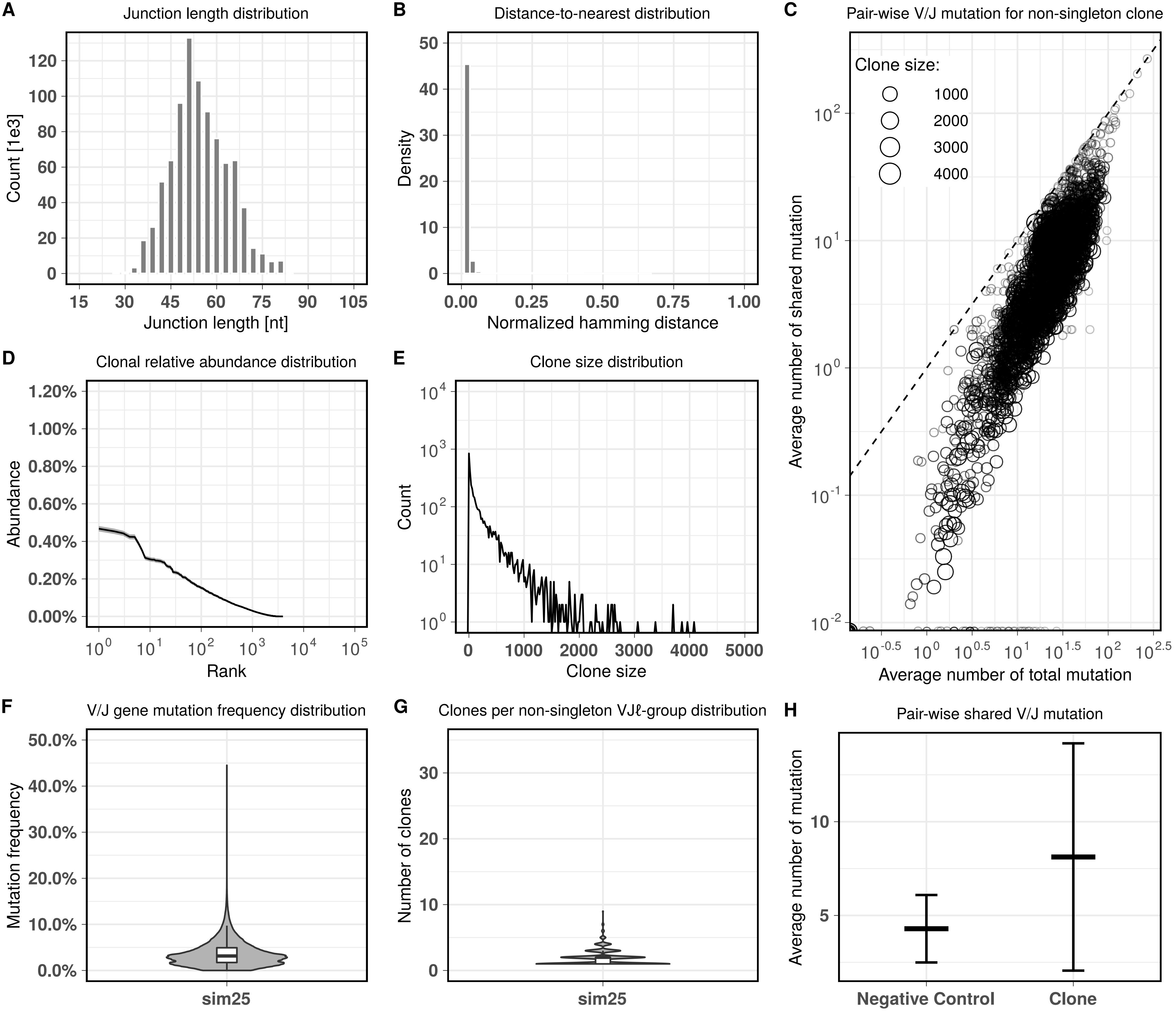

Supplement: S1 Fig — The global metrics of the BCR simulated repertoires, including: (1) junction length distribution, (2) distance-to-nearest distribution, (3) clonal relative abundance distribution, (4) clone size distribution, (5) mutation frequency distribution, (6) number of clones per VJ(ℓ)-group, (7) average pair-wise SHM for clone, and (8) negative-control test (comparing pair-wise SHM sharing rate among real clones and a set of artificial clones generated by randomly sampling sequences across known clones). (PDF) [file pcbi.1007977.s001.pdf]
