# Supplementary material for: Somatic hypermutation analysis for improved identification of B cell clonal families from next-generation sequencing data
Source: PLoS Comput Biol. 2020 Jun 23;16(6):e1007977. doi: 10.1371/journal.pcbi.1007977 (PMC7347241; doi:10.1371/journal.pcbi.1007977)
Supplement: S1 Table — The empirical statistics of BCR experimental repertoires, including: (1) number of total sequences, (2) number of unique sequences, (3) number of inferred clonal lineages, (4) size of largest inferred clonal lineage, (5) number of unique IGHV genes, and (6) number of unique IGHJ genes. (PDF) [file pcbi.1007977.s002.pdf]

Somatic hypermutation analysis for improved identification of B cell clonal families from next-generation sequencing data

Nima Nouri[1,2] and Steven H. Kleinstein[1,2,3,*]

[1]Department of Pathology, Yale School of Medicine, New Haven, CT 06511, USA.
[2]Center for Medical Informatics, Yale School of Medicine, New Haven, CT 06511, USA.
[3]Interdepartmental Program in Computational Biology and Bioinformatics, Yale University, New Haven, CT 06511, USA.

*To whom correspondence should be addressed.
Contact: steven.kleinstein@yale.edu

S1 Table: The empirical statistics of BCR experimental repertoires from 58 individuals with acute dengue infection [*], including: (1) number of total sequences, (2) number of unique sequences, (3) number of inferred clonal lineages, (4) size of largest inferred clonal lineage, (5) number of unique IGHV genes, and (6) number of unique IGHJ genes.

[*] Parameswaran P, Liu Y, Roskin KM, Jackson KK, Dixit VP, Lee JY, et al. Convergent antibody signatures in human dengue. Cell host & microbe. 2013;13(6):691–700.

S1 table: Overview of experimental BCR sequencing data from 58 individuals with acute dengue infection

Subject ID	Sequences	Unique sequences	Clones	Largest clone	Unique V-genes	Unique J-genes
55	7180	6907	5117	168	54	6
97	5408	5061	3350	65	53	6
113	7889	7236	4450	274	56	6
114	6052	5698	4333	56	51	6
128	3726	3238	2262	23	51	6
146	5833	5383	4342	27	52	6
148	1249	1187	364	17	39	6
172	4531	4390	1996	34	51	6
188	2737	2634	1612	15	49	6
189	6666	6244	4526	54	53	6
194	2284	2156	500	26	44	6
199	5368	5012	797	60	49	6
203	5178	5098	3037	40	53	6
208	1762	1628	336	33	37	6
212	8417	6875	3278	54	53	6
232	5500	5426	3461	155	56	6
237	4935	4801	2142	63	51	6
238	6763	6639	3488	278	50	6
240	5249	5136	2475	87	50	6
249	4849	4706	1778	41	48	6
252	3193	3153	2173	32	51	6
255	2490	2468	1727	50	46	6
275	2218	2198	1780	18	47	6
276	2737	2712	2267	11	51	6
287	2773	2724	1593	61	46	6
289	4138	4093	2952	42	52	6
299	3859	3808	2722	23	48	6
301	8174	7939	3534	67	52	6
307	3063	3025	2145	29	51	6
308	5371	4919	3830	17	54	6
311	5031	4954	3159	55	49	6
313	7004	6220	3885	43	56	6
314	9671	8773	4828	723	55	6
320	3219	3189	2089	63	48	6
341	7242	6747	4204	118	53	6
364	6441	5847	4595	19	52	6
376	1552	1528	1037	7	47	6
391	2541	2513	1783	23	51	6
410	9326	8636	3908	290	52	6
422	1253	1239	954	13	47	6
444	3028	3011	2471	96	50	6
455	3282	3255	2711	8	53	6
479	2937	2912	2454	9	48	6
481	2481	2460	2024	14	50	6
489	3019	2978	2339	9	52	6
500	2897	2846	2146	10	52	6
514	2367	2332	1892	5	48	6
515	2534	2469	1603	21	47	6
517	2654	2623	2231	18	48	6
520	5381	5247	2648	103	52	6
524	2943	2911	2184	59	48	6
529	4629	4440	1611	38	51	6
543	2806	2767	2209	26	51	6
551	1351	1328	883	9	44	6
555	4756	4550	2089	68	48	6
558	1836	1800	1179	7	46	6
563	3365	3306	2487	12	51	6
569	1965	1923	1297	10	47	6
